# Is it possible to diagnose the therapeutic adherence of patients with COPD in clinical practice? A cohort study

**DOI:** 10.1186/1471-2466-11-6

**Published:** 2011-01-24

**Authors:** Pilar Barnestein-Fonseca, José Leiva-Fernández, Francisca Vidal-España, Antonio García-Ruiz, Daniel Prados-Torres, Francisca Leiva-Fernández

**Affiliations:** 1 Family and Community Medicine Teaching Unit of Malaga. Health District Malaga. Málaga, Spain; 2 Vélez Sur Health Centre. Axarquía Health District. Vélez Málaga (Málaga), Spain; 3 Sociologist. Family and Community Medicine Teaching Unit of Malaga. Health District Malaga. Málaga, Spain; 4 Farmacoeconomy and SRI Unit. Farmacoeconomy and Clinical Therapeutic Department. Faculty of Medicine. Malaga University. Málaga, Spain; 5 Family and Community Medicine Teaching Unit of Malaga. Health District Malaga. Málaga, Spain; 6 Family and Community Medicine Teaching Unit of Malaga. Health District Malaga. Málaga, Spain

## Abstract

**Background:**

Therapeutic adherence of patients with chronic obstructive pulmonary disease (COPD) is poor. It is therefore necessary to determine the magnitude of non-adherence to develop strategies to correct this behaviour. The purpose of this study was to analyse the diagnostic validity of indirect adherence methods.

**Methods:**

Sample: 195 COPD patients undergoing scheduled inhaled treatment attending 5 Primary Care Centres of Malaga, Spain. Variables: Sociodemographic profile, illness data, spirometry, quality of life (St. George Respiratory Questionnaire: SGRQ), and inhaled medication counting (count of dose/pill or electronic monitoring) were collected. The patient's knowledge of COPD (Batalla test:BT),their attitude towards treatment (Morisky-Green test: MGT) and their self-reported therapeutic adherence (Haynes-Sackett test: HST) were used as methods of evaluating adherence. The follow-up consisted four visits over one year (the recruitment visit: V0; and after 1 month:V1; 6 months:V2; and 1 year:V3).

**Results:**

The mean age was 69.59 (95% CI, 68.29-70.89) years old and 93.8% were male. Other findings included: 85.4% had a low educational level, 23.6% were smokers, 71.5% mild-moderate COPD stage with a FEV1 = 56.86 (SD = 18.85); exacerbations per year = 1.41(95% CI, 1-1.8). The total SGRQ score was 44.96 (95% CI, 42.46-47.46), showing a mild self-perceived impairment in health. The prevalence of adherence (dose/pill count) was 68.1% (95% CI, 60.9-75.3) at V1, 80% (95% CI, 73-87) at V2 and 84% (95% CI, 77.9) at V3. The MGT showed a specificity of 67.34% at V1, 76.19% at V2 and 69.62% at V3. The sensitivity was 53.33% at V1, 66.66% at V2 and 33.33% at V3.The BT showed a specificity of 55.1% at V1, 70.23% at V2 and 67.09% at V3. The sensitivity was 68.88% at V1, 71.43% at V2 and 46.66% at V3. Considering both tests together, the specificity was 86.73% at V1, 94.04% at V2 and 92.49% at V3 and the sensitivity was 37.77% at V1, 47.62% at V2 and 13.3% at V3.

**Conclusions:**

The prevalence of treatment adherence changes over time. Indirect methods (dose/pill count and self-reported) can be useful to detect non-adherence in COPD patients. The combination of MGT and BT is the best approach to test self-reported adherence.

## Background

Chronic obstructive pulmonary disease (COPD) is currently the fourth leading cause of death world-wide. Furthermore, the prevalence of COPD is increasing and it is estimated that by 2020 COPD will be the third leading cause of death [1,2].

Important progress has been made in the pharmacological and non-pharmacological treatment of COPD. While a major goal of therapy is to provide symptoms relief, only the effective management of COPD has been shown to reduce the rate of exacerbations, hospitalisations and mortality and to improve health-related quality of life [3]. The effectiveness of treatment relies on patient agreement with adherence to the therapy regime.

As with all chronic diseases, non-adherence in patients with COPD is common and contributes to adverse health outcomes, reduced quality of life and increased healthcare expenditure [4]. According to the World Health Organization, patient adherence to long-term therapy averages 50% [5]. In the Lung Health Study [6], therapeutic adherence with inhaled treatment recorded by self-reported methods after one year of follow-up was 60%, decreasing to 50% after five years of follow-up.

Different types of non adherence exist in COPD patients [7], including underuse (a reduction in the apparent daily use versus the standard dose of a medication that is indicated for the treatment or prevention of a disease or condition), overuse (use of higher than prescribed treatment doses, or shorter intervals between doses), improper or inappropriate use (the drug is ineffective, not indicated or if there is unnecessary duplication of therapy).

The most common type of non-adherence in COPD patients is underuse [8], and improper use is the most frequent type of non-adherence in patients older than 65 years of age with polypharmacy. In COPD, underuse is followed in frequency by overuse and improper use of the medication-delivering device. Underuse can be sporadic or systematic, from forgetting an occasional dose to changing dosing schedules; patients with underuse are at a higher risk for adherence-related morbidity [7].

Several methods exist to measure adherence in COPD, each with its own strengths and limitations. Biochemical evaluation of drug level can confirm the intake. However, this method is both expensive and invasive and can also reflect other factors, resulting in pharmacokinetic variations [9]. Electronic monitors are increasingly being used in clinical trials to measure adherence. They provide accurate and reliable records of dosage but are expensive, subject to malfunction and cannot confirm ingestion [10]. Other common methods to measure adherence include patients' diaries, pill counts, canister weighing and analysis of computerized pharmacy records, but these can overestimate adherence [11]. Pill count is limited to oral medications and only assesses whether the correct number of pills have been removed, it does not indicate ingestion, dose or dose frequency. However, this method is simple, available and useful in daily clinical practice as an approach to measuring adherence. Likewise, canister weighing is not reliable because activation of the device immediately prior to the visit can suggest adherence. Analysis of pharmacy records provides evidence of drug refill patterns but cannot assess ingestion or pattern of use [3].

The easiest approach to assessing adherence is to simply query the patient, but this method generally overestimates medication use [4,12]. Physician assessment of their patients' adherence is similar [13]. In the clinical setting, therefore, reliance on any single method of assessment can be misleading.

On the other hand, patients who are not adherent and who are aware of this behaviour are those who are going to respond better to health educational policies. So, it is necessary to identify them properly and this would probably allows us to form patients groups more effectively for future educational interventions.

Nevertheless, it is still necessary to determine the magnitude of non-adherence in COPD patients as the first step towards developing strategies to correct these behaviours.

The objective of this study was to asses the diagnostic validity to estimate the prevalence of non-adherence in COPD patients with inhaled medication using three self-reported methods that could be useful and easy in clinical practice, considering the electronic monitoring or the pill count as the reference method.

## Methods

**Figure 1:**
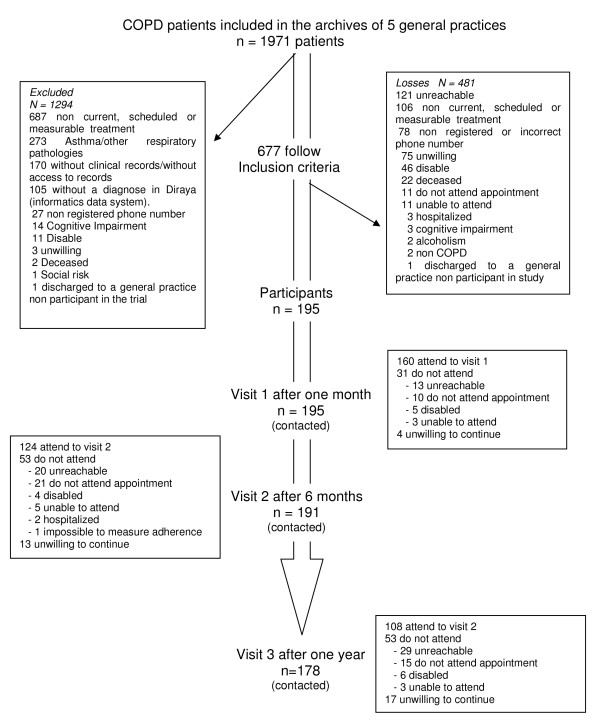
**Study general graphic**.

A cohort study was undertaken in 5 Health Care Center in Málaga Province. The subjects were COPD patients and with a medical records contained data about the diagnosis and treatment of their disease. The recruitment period was six months and the follow-up period was one year (Figure 1). The inclusion criteria were: a diagnosis of COPD in the patient's clinical record, taking scheduled inhaled therapy, and belonging to the basic health care area. The exclusion criteria were: patients with any respiratory condition not included in the definition of COPD (bronchiectasis, asthma or cystic fibrosis) and patients with cognitive impairment.

The protocol and the patient information form were approved by a primary care ethics committee, and all the patients signed informed consent forms before their inclusion in the study.

We calculated the sample size according to the following data: (1) a prevalence of non-adherence using the MGT of 65%, found in a previous study of a cohort of COPD patients performed by our group (data not published); (2) 95% confidence level; (3) 7% precision and (4) 35% of expected losses. The final sample size was calculated to be 237 participants, who were then selected by a non-probabilistic sampling method.

Sociodemographic variables (age, sex, civil status, educational level); Body mass index (BMI); COPD-related variables (smoking habits, number of cigarettes, treatment and exacerbations); forced spirometry according to the SEPAR guidelines [14]; comorbidity and quality of life using the St George Respiratory Questionnaire (SGRQ). The Spanish version of the SGRQ [15,16] is a specific instrument for measuring quality of life in patients with respiratory disease. It has 50 items divided into three scales: symptoms (frequency and severity of respiratory symptoms), activity (activity limitations due to dyspnoea) and impact (psychological and social disorders due to respiratory disease). SGRQ scores range from 0-100, with zero indicating no impairment in quality of life. The SGRQ can be applied by a self-administered way or it can be read to the subject if he has reading impairments. We selected the second way because of the low social and cultural level of the study group.

### Adherence

Adherence to (or compliance with) a medication regimen is generally defined as "the extent to which a person's behaviour -taking medication, following a diet and/or executing lifestyle changes- corresponds with agreed recommendations from a health care provider" [17]. Adherence to inhaled therapy was measured by dose/pill count or electronic monitoring. This was considered to be the reference method, and was performed at an unexpected appointment at the health centre. An electronic dose counter (DOSER [18]) was used to recount the metered dose inhaler (MDI) devices, and doses or pills were counted in the other inhaler devices.

Adherence was measured as the number of pills or doses taken divided by the number of pills or doses prescribed, multiplied by 100 (expressed as a percentage). In accordance with Sackett et al. [19] recommendations, a good adherence is considered when the result of counting is between 80% (20% loss of doses/pills) and 110% (the patient consumes 10% more doses/pills) of doses/pills prescribed. This cutoff point was selected for consistency with other studies [20].

### Self-reported adherence methods

Three self-reported methods were selected to evaluate treatment adherence: the Haynes and Sackett method [21], the Morinsky Green test [22] and the Batalla test [23]. These questionnaires to assess adherence are normally used for chronic conditions and have been adapted and validated for the Spanish population for conditions such as hypertension and hyperlipidaemia [24,25].

#### Haynes and Sackett method (HST)

Self-reported assessment of adherence was introduced in the following sentence: "People often have difficulty taking their pills for one reason or another and we are interested in finding out any problems that occur so that we can understand them better." Patients were then asked whether they ever missed their pills and, if so, to state their current prescriptions and the average number of tablets missed per month [19]. Good adherence was considered to be when the percentage of doses taken was between 80% and 110% of the prescribed dose.

#### Morinsky Green test (MGT)

We measured the attitude towards treatment using the MGT [22], adapted by us for use with inhaled medication: (1) Do you ever forget to take your inhaled medication? (2) Are you careless at times about taking your inhaled medication? (3) When you feel better, do you sometimes stop taking your inhaled medication? (4) Sometimes, if you feel worse when you take the inhaled medication, do you stop taking it? We considered good adherence to be when all four questions were answered suitably.

#### Batalla test (BT)

The BT provides information about the patients' understanding of their illness [23]. The questions, adapted to COPD, used in this study were as follows: (1) Is COPD a lifelong disease?, (2) Can you control this disease by quitting smoking and/or with medication?, (3) Mention one or more organs that can get damaged by COPD. We considered good adherence to be when the patient was able to answer these three questions suitably.

### Recruitment and follow-up

Each Health Care Center provided the list of patients included in the COPD Health Program. Once we identified the possible candidates, they were invited by phone to participate in the study and given an appointment at their primary health care centre. At this appointment (**recruitment visit: V0**) we gave the patients a fuller explanation of the study and they signed the informed consent form prior to inclusion. After inclusion we measured all the study variables.

In addition, at this first visit we provided the patients with a new MDI with an electronic dose counting device or a new device for pill/dose count, and the patients were told they would be phoned again for review but they were not told when this would take place. The patients were given another appointment after one month (**V1**) and the treatment adherence was evaluated by electronic monitoring or pill/dose count along with the self-reported adherence tests.

The participants were again contacted five months after the first visit and a new MDI with a dose counting device or a new device for pill/dose count was provided. The count was done one month later (**6 months visit: V2**), along with self-reported adherence tests. We also measured other variables changes.

Finally, 11 months after starting the study, the patients were given an appointment in order to receive another new counting device, which was read the following month (**one year visit: V3**), along with self-reported adherence tests, forced spirometry, SGRQ and measurement of the other variables.

### Statistical analysis

A descriptive analysis was made of all the study variables, calculating the mean, median, standard deviation, total frequency and relative frequency of each category; 95% confidence intervals were calculated for the means and proportions. We considered dose/pill count to be the reference method for assessing adherence. We performed two types of analytical strategies [26] to compare the reference value and the self-reported methods in order to evaluate their validity to diagnose adherence: 1) open comparison to explore the existence of a statistical association between each self-reported questionnaire and the reference method using the Chi-square test, and 2) hierarchy comparison in which we assumed that the reference method is the best method to assess non therapeutic adherence, and we then calculated the kappa value (as a measure of agreement between the reference method and each self-reported test), the basic diagnostic descriptors (sensitivity and specificity) and their combination (likelihood ratio) for each of the self-reported methods. To achieve this we elaborated 2 × 2 tables and calculated the following indicators of diagnostic validity for each test: Sensitivity = true positive/(true positive+false negative); Specificity = true negative/(true negative+false positive); Positive likelihood ratio = sensitivity/(1-specificity); Negative likelihood ratio = (1-sensitivity)/specificity. The data analysis was carried out using the statistical package SPSS/PC, version 15.0.

## Results

### Sample characterization

#### Baseline

The main reasons for losses were, firstly, the difficulties experienced in the review of the clinical records; the clinical records of 1971 COPD patients were reviewed but we found many false positive COPD diagnoses as well as other respiratory conditions classified as COPD. Secondly, the strict inclusion criteria used in this study, such as the use of inhalers with a dose counter or that could use a DOSER device. In addition, we were unable to contact many patients and others refused to participate in the study (Figure 1).

The final sample consisted of 195 patients with a diagnosis of COPD (Table 1), predominantly male (93.8%), with a mean age of 69.59 years (95% CI, 68.29-70.89 years), and with a low educational level (50% with no studies). At the time of the study, 23.6% were active smokers, with a mean of 17.52 cigarettes per day (95% CI, 14.34-20.7) and many were overweight [mean BMI 29.55 (95% CI, 28.87-30.23)]. Concerning the COPD severity stage, 71.5% were classified as mild to moderate, with a mixed spirometric pattern (62.5%), and a mean FEV1 of 56.86 (SD:18.85). More than half the patients had experienced at least one exacerbation in the last year [mean exacerbations 1.41 (95% CI, 1-1.8)].

**Table 1:** Sociodemographic and clinical profile.

Number of subjects	195
Gender	
Male	93.8%
Age (mean, 95% CI)	69.59 (68.29-70.89)
Education levels	
Without education	50%
Primary education	35.4%
Secondary education	9.4%
Higher education	4.7%
Civil Status	
Single	3.6%
Married	85.6%
Widowed	8.7%
BMI (mean, 95% CI)	29.55 (28.87-30.23)
Smoking habits	
Non smokers	7.2%
Smokers	23.6%
Ex smokers	69.2%
N° exacerbations (mean, 95% CI)	1.41 (1-1.8)
N° visits at PCC	4.78
N° visits due to COPD	2.06

IC: Confidence interval

PCC: Primary Care Center

##### Drug therapy

**Figure 2:**
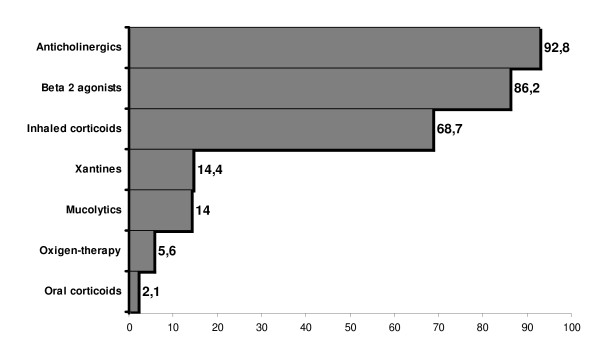
**Prescribed treatment for COPD patients (numbers in bars expressed percentage of patients with the indicated therapeutic group prescribed)**.

The treatment prescribed for their condition is shown in Figure 2 (represented as the percentage of patients receiving a particular treatment). We see that 92.8% of the patients were taking anticholinergic drugs, 47% short-acting and 74% long-acting, prescribed either individually or jointly according to the cases. Beta-2 agonists were prescribed for 86.2% of the patients, 68.45% of these were short-acting and 77.38% long-acting. Inhaled corticosteroids were prescribed for 68.7% of the patients, in most cases combined with a beta-2 agonist. Figure 2 also provides information about the other drugs. Most patients (89.6%) were prescribed combined therapy from more than one pharmacological group. The most frequent treatment was the combination of 3 groups (46.6%): an anticholinergic drug with a long-acting beta-2 adrenergic agonist and an inhaled corticosteroid.

##### Quality of life

The SGRQ scores were: symptoms scale, 46.03 (95% CI, 43.25-48.81); activity scale, 61.2 (95% CI, 58.15-64.25); impact scale, 35.34 (95% CI, 32.56-38.12); and total score, 44.96 (95% CI, 42.46-47.46).

##### Adherence measures

Self-reported methods were also used at the first inclusion visit, observing variability in the outcomes. This enabled us to determine that the percentage of patients with good adherence was 99.5% with the HST, 55.9% with the BT, and 52.1% with the MGT.

#### Follow-up

**V1**: 160 patients (82%) out of the 195 included in the study attended the first control visit one month after inclusion. The losses at follow-up and their reasons are shown in Figure 1. Of the 160 patients evaluated, 13 changed their therapeutic procedure, using inhalers when needed with no scheduled treatment; these patients were thus excluded from the analysis. A change in prescription occurred in 8.1% of the patients, half of them due to addition of another medicine, mostly because of an exacerbation or a cold, and 37.27% because of a dose modification. At least one exacerbation in the month following the inclusion visit was experienced by 18.8% of the patients.

**V2**: 124 (63.5%) out of the 195 included in the study attended the second control visit after 6 months (Figure 1). Of the 124, eight changed their therapeutic procedure, using inhalers when needed with no scheduled treatment; these patients were thus excluded from the analysis. A change in treatment was made for 26.2% of the patients, 40.45% due to the addition of a new drug and 34.35% due to a dose change. At least one exacerbation in the 6 months following the inclusion visit was experienced by 31.7% of the patients.

**V3**: 108 (55.4%) out of the 195 included in the study attended the third control visit one year after inclusion (Figure 1). Of the 108, 38.6% experienced a change in their prescription, most frequently concerning the addition of a new drug (26.68% of the cases), followed by a dose change (21.24%) and total treatment renewal (13.21%). At least one exacerbation since the previous follow-up visit was experienced by 35.7% of the patients.

##### Treatment adherence

**V1**: Adherence prevalence using the reference method was 68.1%. When we assessed the self-reported adherence methods, these were 100% for the HST, 60.8% for the MGT and 46.9% for the BT. The MGT detected 24 of the 45 patients classified as non-adherent by RM method, while the BT found 31. Considering both tests together, 38 out of the 45 non-adherent patients were detected. The chi-square test showed a significant association between the reference method and the self-reported methods (Table 2). The measure of agreement by kappa (*k*) between MGT and the reference method was 0.194 (p = 0.019), for the BT *k *= 0.194 (p = 0.011), and when we considered both tests together *k *= 0.143 (p = 0.019).

**Table 2:** Open comparison of adherence prevalence between the self-reported methods and the reference method by the chi-square test.

	1 month	6 months	12 months
**Dose/pill count**	**68.1% (60.9-75.3)**	**80% (73-87)**	**84%(77-90)**

Morinsky-Green Test	60.8% (53.3-68.3) *p = 0.019*	67.6% (59.4-75.8) *p < 0.001*	69.1% (60.4-77.8) *p = 0.820*

Batalla Test	46.9% (39.2-54.6) *p = 0.011*	61.9% (53.4-70) *p < 0.001*	64.9% (55.9-73.9) *p = 0.306*

Morinsky-Green Test + Batalla Test	28.7% (21.7-35.7) *p = 0.002*	43.8% (35.1-52.5) *p < 0.001*	42.6% (33.6-51.6) *p = 0.633*

**V2**: We observed an adherence prevalence of 80% evaluated by the reference method and of 100%, 67.7% and 61.9% for the HST, the MGT and the BT. The MGT detected 14 of the 21 patients with non-adherence, while the BT found 15. Considering both tests together, 19 out of the 21 patients with non-adherence were detected. The chi-square test showed a significant association between the reference method and the self-reported methods (Table 2). The measure of agreement by kappa (*k*) between MGT and the reference method was 0.348 (p = 0.001), for the BT *k *= 0.311 (p = 0.001) and when we considered both tests together *k *= 0.255 (p = 0.001).

**V3**: We observed an adherence prevalence of 84% evaluated by the reference method. When we assessed the indirect adherence methods, these were 100% for the HST, 64.1% for the MGT and 64.9% for the BT. The MGT detected 5 of the 15 patients with non-adherence, while the BT found 7. Considering both tests together, 10 out of the 15 patients with non-adherence were detected. The chi-square test showed no significant association between the reference method and the self-reported methods at this visit (Table 2). The measure of agreement by kappa (*k*) between MGT and the reference method was 0.021 (p = 0.82), for the BT *k *= 0.093 (p = 0.30) and when we considered both tests together *k *= 0.053 (p = 0.43).

**Figure 3:**
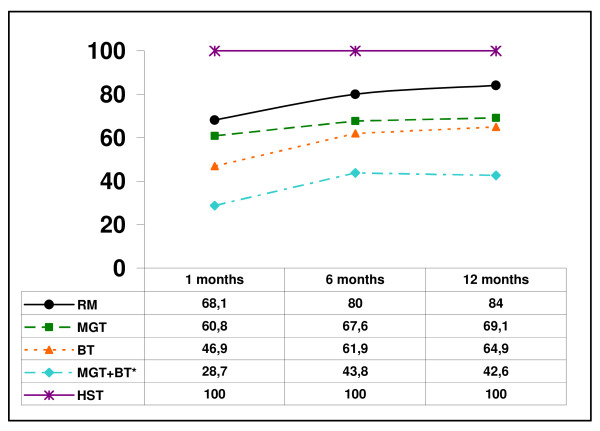
**Evolution of the adherence treatment percentage after one year of monitoring**. %patients. RM: dose/pill count; MGT: Morinsky-Green Test; BT:Batalla test;HST: Haynes- Sackett Self-report. * Adherent patients diagnosed by both methods.

Figure 3 shows the course of inhaled treatment adherence over time. We can see an increase of adherence along the study in all indirect methods used.

Table 3 shows the diagnostic validity characteristics of the two adherence evaluation methods.

**Table 3:** Diagnostic validity of self-reported methods to detect non adherent patients with the prescribed treatment.

	VISIT 1	VISIT 2	VISIT 3
**Non adherence (reference method)**	31.9%	20%	16%

			

***Morisky-Green Test***			

Non adherence	39.2%	32.4%	30.9%

Sensitivity	53.33% (38.76-68)	66.66%(46.66-86.66)	33.33%(10.3-56.3)

Specificity	67.34% (58.06-76.62)	76.19% (67-85.3)	69.62%(59.6-79.6)

Positive Predictive Value	42.86%	41.17%	17.24%

Negative Predictive Value	75.86%	90.14%	84.61%

Positive Likelihood Ratio	1.66	2.79	1.09

Negative Likelihood Ratio	0.69	0.43	0.95

			

**Batalla Test**			

Non adherence	53.1%	38.1%	35.1%

Sensitivity	68.88%(55.36-82.4)	71.43%(52.1-90)	46.66%(21.6-71.66)

Specificity	55.1%(45.3-64.9)	70.23%(60.5-80)	67.09%(57.09-77.09)

Positive Predictive Value	40.79%	37.5%	21.21%

Negative Predictive Value	85.07%	90.77%	86.88%

Positive Likelihood Ratio	1.53	2.38	1.41

Negative Likelihood Ratio	0.56	0.4	0.79

			

**MGT-BT**^**1- **^**(non.adherent at least by one of two methods)**			

Non adherence	71.3%	56.2%	57.4%

Sensitivity	84.44%(74-95)	90.47%(78.47-100)	66.66%(56.66-76.66)

Specificity	34.69%(25.3-44.1)	52.38%(42.38-62.38)	44.3%(34.3-54.3)

Positive Predictive Value	37.25%	32.2%	18.51%

Negative Predictive Value	82.92%	95.6%	87.5%

Positive Likelihood Ratio	1.29	1.89	1.19

Negative Likelihood Ratio	0.45	0.18	0.75

**MGT-BT**^**2- **^**(non-adherent by the two methods)**			

Non adherence	21%	14.3%	8.5%

Sensitivity	37.77%(23.6-52)	47.62%(26.6-68.6)	13.3%(0-30)

Specificity	86.73%(80-93.4)	94.04% (89-99)	92.49%(86.6-98.2)

Positive Predictive Value	56.66%	66.66%	25%

Negative Predictive Value	82.52%	87.77%	84.88%

Positive Likelihood Ratio	2.84	7.98	1.77

Negative Likelihood Ratio	0.71	0.55	0.93

Morinsky-Green Test : MGT; Batalla Test:BT; Sensitivity : S ; Specificity : E ; Positive Probability Ratio: PPR.

## Discussion

The diagnostic instruments [27] used in medicine have traditionally been considered as a mean to reduce diagnostic uncertainty. This uncertainty reaches important levels if we are trying to diagnose an entity such as treatment adherence, which is influenced by a great diversity of factors. As treatment adherence has to be quantified with the best test available, it is necessary to evaluate which methods are most suitable for clinical monitoring and then measure the indicators of diagnostic validity [24,25], selecting a reference method that should be considered as a gold standard.

In the current study we aimed to review all these points, centred on patients included in COPD care programs in general practice setting. The first difficulty encountered was the high percentage of patients included in the COPD records with an incorrect diagnosis or the lack of data necessary to locate the patients. These situations accounted for most of the losses at the start, as well as during the monitoring period of the trial. Nevertheless, no significant differences were found between the losses and those who were included, so we can conclude that the main effect of these losses is probably related with statistical power. The final study sample was similar to that described by others, except for a higher mean age and a lower educational level [28,29]. We still have a high prevalence in male sex probably due to the smoking habit in our area. Patients with mild to moderate COPD stages are predominant in the sample because SEPAR guidelines [30] recommend the management of these patients at primary care setting and the management of patients with severe and very severe COPD by the Neumologist. Patients with mild-moderate COPD predominated in our sample, most of them receiving medical treatment with anticholinergic and beta-2 agonist drugs, as recommended at this stage of the disease [30,31].

By telephoning the patient unexpectedly one day before the appointment at the health centre to review adherence we attempted to avoid any behavioural changes that could affect treatment adherence in the days before the appointment.

For patients with chronic diseases seen in the primary care setting, the dose/pill count or electronic monitoring at the general practice have been used as the reference method to define the diagnostic validity of the self-reported methods [24,25,32-34], although it was found that 22% of patients with hypertension who opened the electronic monitoring (EM) containers did not take sufficient drugs and 35% who seemed to take their pills did not open the EM container as frequently as prescribed [33]. The main disadvantage of this method is that it assumes that every missing dose/pill from the package has been taken by the patient. Accordingly, we can consider that it overestimates the prevalence of treatment adherence. However, the dose/pill count and EM are simple, available and less expensive than others methods. This is why we used the dose/pill count as the reference method in our study. No perfect reference method (gold standard) exists owing to the nature of the process that we are studying, so we assume we have made the imperfect gold standard bias [26]. The use of an electronic counter for MDI in COPD is usual [3,4,6], but in fact the measure of adherence is more frequently obtained by self-reported methods [3,7].

The adherence prevalence in our study, with the dose/pill count, was similar to that found in other studies [35-37], although in these studies the measure of adherence was made by different methods: canister weight [35], pharmacy database medication refill [36], or self-reported methods [37]. We detected an improvement on adherence prevalence over time that could be explained by two features. The first is the Hawthorne effect along the study (ie, tendency of subjects participating in a research study to change their behaviour). Although this could affect overall estimates of adherence, the implications might be less important in comparing results of different measurements tools (unless, of course, the effect is differentially captured by each measurement tool); furthermore, it is difficult to perceive that any potential Hawthorne effect would be maintained over the many months of study. The second one is related with a sample self-selection, where the better adherent patients were who continued in the study over time.

Three instruments were selected in the present study as methods of evaluating self-reported of adherence. These methods have been validated in chronic disorders other than respiratory diseases [24,25,33,34].

Comparison of the prevalence found using the reference method with the estimates of the self-reported methods shows that self-reported adherence using the HST overestimated the adherence percentage, while the MGT and the BT produced lower compliance values. Although self-reported compliance using the HST has shown an adequate diagnostic validity in other trials with chronic patients [23,24], in our COPD study it overestimated adherence and showed no changes over time (as can be seen in Figure 3), since it classified as adherent 100% of the patients at all three evaluations made. Therefore, we do not consider it suitable to use for this disease.

The open comparison of adherence prevalence between the self-reported methods and the reference method (Table 2) shows a significant association (p < 0.05) at V1 and V2. No significant association was seen at V3, possibly due to the low number of non-adherent patients.

In order to determine the suitability of the other two methods, we measured their agreement by kappa. The agreement between the reference method and the self-reported methods was poor at V1 (*k *= 0.143-0.194) and fair at V2 (*k *= 0.255-0.348). Thus, although there is agreement between the two measures of adherence, the concordance was low. However, no kappa value can be universally regarded as indicating good agreement, and this value cannot substitute clinical judgement [38]. In addition, the kappa value depends on the proportion of subjects in each category [38]. In our case this characteristic of kappa was very important because of the great difference between the proportion of adherent and non-adherent subjects during the follow-up. At V1 and V2 the proportion difference was 2:1, but at V3 this difference was more than 4:1. In this situation the calculated kappa value can be misleading and we have to consider the clinical context for its interpretation.

The MGT shows the attitude towards treatment, the BT shows the understanding of the illness and the reference method informs us about the action of taking the treatment. We think that the measures of adherence all assess the same behaviour, though with a few differences, i.e., the focus, the question itself and the different components of behaviour. These differences could, we believe, explain the poor magnitude for agreement.

The diagnostic validity indicators obtained by the other two self-reported methods (MGT and BT) considered independently show a sensitivity and specificity fluctuating between 33.33% and 66.66% for the MGT and between 47% and 69% for the BT. These values changed when we considered both tests together (both tests classify the patient as non-adherent), observing a considerable increase in the specificity, which varied between 86% and 94%, with a reduction in sensitivity (13-47%). These values match those of other trials for chronic diseases, in which the specificity surpasses sensitivity [24,25]. In our case this means that we would classify correctly adherent subjects (true negative) because sensitivity is low and specificity is high. In clinical practice this is very useful because when you use both tests with a patient and he or she is classified as adherent you can be sure that this is the case.

If we consider the likelihood ratio to detect non-adherent patients we see that it varied between 2.84 at V1 and 7.98 at V2 (when the non-adherence prevalence was 21%), which means that when the result of both tests identifies a patient as non-adherent with the scheduled inhaled treatment, it is nearly 8-fold more likely to be a true positive value. At V3 (when the non-adherence prevalence was 16%), the likelihood ratio decreased to 1.77. This result suggests that there is a critical point of non-adherence prevalence below which the tests lose their ability to detect non-adherent behaviour with sufficient reliability.

Considering all the results of the study, we propose a practical approach to assess self-adherence in clinical practice that depends on the expected adherence prevalence:

- If we expect a high or medium non-adherence prevalence in our clinical setting, we can use the self-reported methods (MGT and BT together) to detect and to follow-up the non-adherent patients. We can thus detect non adherence in a simple and rapid manner.

- If we expect a low non-adherence prevalence, we can use the dose/pill count or EM for MDI devices.

## Conclusion

Despite the fact that methods to measure adherence are not perfect, it is better to use them in a homogeneous and structured manner rather than not to take them into account. The dose/pill count could be chosen in clinical practice, even though we know that it overestimates adherence. An alternative to the dose/pill or EM count is a self-reported method, but the diagnostic validity of the two tests performed independently is low. Nevertheless, when they are considered together they have a higher potential to detect patients with non-adherence to therapeutic regimens and at a low cost.

Despite the limitations of this study, we nevertheless consider that it gives us more information with which to improve the quantitative evaluation of adherence by the COPD patient. However, an important problem still remains to be resolved: the incorrect use of the different inhaler devices, even in compliant patients, in whom it should also be evaluated.

## Competing interests

The authors declare that they have no competing interests.

## Authors' contributions

PBF was involved in drafting the manuscript and writing it. She participated in coordination of the study, recruitment and follow-up of the patients and statistical analysis and interpretation of data. JLF was involved in recruitment and follow-up of the patients, interpretation of data and writing the manuscript. FVE was involved in recruitment and follow-up of the patients, interpretation of data and writing the manuscript. AGR was involved in the design and coordination of the study, and statistical analysis and interpretation of data. Finally he participated in writing the manuscript. DPT was involved in the design and coordination of the study, and interpretation of data. Finally he participated in writing the manuscript. FLF was involved in writing the manuscript. She participated in the design and coordination of the study, and statistical analysis and interpretation of data. All authors have read and approved the final version of the manuscript.

## Pre-publication history

The pre-publication history for this paper can be accessed here:

http://www.biomedcentral.com/1471-2466/11/6/prepub
